# Global changes in gene expression associated with phenotypic switching of wild yeast

**DOI:** 10.1186/1471-2164-15-136

**Published:** 2014-02-17

**Authors:** Vratislav Šťovíček, Libuše Váchová, Markéta Begany, Derek Wilkinson, Zdena Palková

**Affiliations:** 1 Department of Genetics and Microbiology, Faculty of Science, Charles University in Prague, Viničná 5, 128 44 Prague 2, Czech Republic; 2 Institute of Microbiology of the ASCR, v.v.i., Vídeňská 1083, 142 20 Prague 4, Czech Republic; 3 Current Address: The Novo Nordisk Foundation Center for Biosustainability, Technical University of Denmark, Kogle Alle 6, 2970 Hørsholm, Denmark

**Keywords:** Biofilm colony, Histone deacetylase, Phenotypic switching, Wild yeast strains

## Abstract

**Background:**

*Saccharomyces cerevisiae* strains isolated from natural settings form structured biofilm colonies that are equipped with intricate protective mechanisms. These wild strains are able to reprogram themselves with a certain frequency during cultivation in plentiful laboratory conditions. The resulting domesticated strains switch off certain protective mechanisms and form smooth colonies that resemble those of common laboratory strains.

**Results:**

Here, we show that domestication can be reversed when a domesticated strain is challenged by various adverse conditions; the resulting feral strain restores its ability to form structured biofilm colonies. Phenotypic, microscopic and transcriptomic analyses show that phenotypic transition is a complex process that affects various aspects of feral strain physiology; it leads to a phenotype that resembles the original wild strain in some aspects and the domesticated derivative in others. We specify the genetic determinants that are likely involved in the formation of a structured biofilm colonies. In addition to *FLO11*, these determinants include genes that affect the cell wall and membrane composition. We also identify changes occurring during phenotypic transitions that affect other properties of phenotypic strain-variants, such as resistance to the impact of environmental stress. Here we document the regulatory role of the histone deacetylase Hda1p in developing such a resistance.

**Conclusions:**

We provide detailed analysis of transcriptomic and phenotypic modulations of three related *S. cerevisiae* strains that arose by phenotypic switching under diverse environmental conditions. We identify changes specifically related to a strain’s ability to create complex structured colonies; we also show that other changes, such as genome rearrangement(s), are unrelated to this ability. Finally, we identify the importance of histone deacetylase Hda1p in strain resistance to stresses.

## Background

In the natural environment, microorganisms do not exist in stable and optimal growth conditions. Instead, microbial populations are often challenged by harmful external factors. The formation of complex multicellular communities, such as biofilms or colonies, provides an advantage for survival in the wild due to the cooperative behavior of cells and the establishment of common protective mechanisms [1]. Moreover, the adaptive responses of individual cells and their phenotypic heterogeneity, factors that enable efficient adaptation to a rapidly changing environment, are also important characteristics that allow populations to thrive in natural settings. Phenotypic switching is a mechanism by which phenotypic heterogeneity is achieved. This process generates cell phenotypes that are better adapted to a particular environment, thereby allowing a population to react flexibly to environmental changes [2]. In yeast, phenotypic switching generates a diverse array of changes, leading to the emergence of colonies with altered morphologies [3]. The appearance of different colonies occurs more frequently than somatic mutations. Although relatively stable during subsequent passages, the switched variants retain the ability to revert to their original phenotype [4].

Phenotypic switching is a phenomenon that is commonly observed in pathogenic yeasts [5]. Switching occurs at infection sites to generate phenotypes that effectively evade the host immune response [6]. The new phenotypes also exhibit features that contribute to increased virulence [7,8]. A phenotypic switch is defined as a random event that is not necessarily induced by external factors; however, environmental conditions can affect the frequency and direction of the switch. For example, certain strains of *Candida albicans* undergo a transition from a “white” to “opaque” colony phenotype and vice versa. Exposure to temperatures above 30°C increases the frequency of switching to the “white” phenotype [9]. Thus, white cells predominate in the blood stream at a physiological temperature of 37°C, whereas “opaque” cells more efficiently colonize the skin surface, an environment with a lower temperature [7]. Furthermore, anaerobic conditions and other environmental factors also mediate the transition to the “opaque” phenotype [10]. Thus, switching provides a mechanism of adaptation to certain niches and to a variety of physiological conditions.

Wild *Saccharomyces cerevisiae* strains isolated from their natural habitat also exhibit phenotypic heterogeneity and the ability to switch between two or more different colony phenotypes [11-15]. During laboratory cultivation on rich media, the switch is usually oriented toward the formation of less-structured colonies that differ in many features from their structured counterparts [14]. Such a switch, that we term “domestication” [13] results in the formation of strains that are stable during subsequent passages on agar media and that form smooth colonies similar to those formed by standard *S. cerevisiae* laboratory strains [13]. The formation of a domesticated derivative, BR-S, of the wild *S. cerevisiae* BR-F strain on a non-fermentable medium occurs efficiently with an average frequency of approximately 2-3% [13]. This frequency suggests that switching is caused by a regulated event rather than by random mutations. During cultivation of a BR-S strain under adverse conditions, we demonstrate the occurrence of reverse phenotypic changes that lead to the re-appearance of cell clones that form more structured colonies. We termed these strains “feral” strains. By comparing the transcriptomes of the original wild BR-F strain, its domesticated BR-S derivative and the feral BR-RF strain (the strain that restored biofilm colony formation), we determine the genome-wide expression alterations involved in phenotypic changes. In addition, we specify the factors involved in the formation of structured biofilm colonies. Finally, we show that at least some individual strain properties are under the control of epigenetic mechanisms and that recombination and genome rearrangements occur during phenotypic switching.

## Results

### Feral subclones derived from a BR-S strain under stress conditions form structured colonies

**Figure 1:**
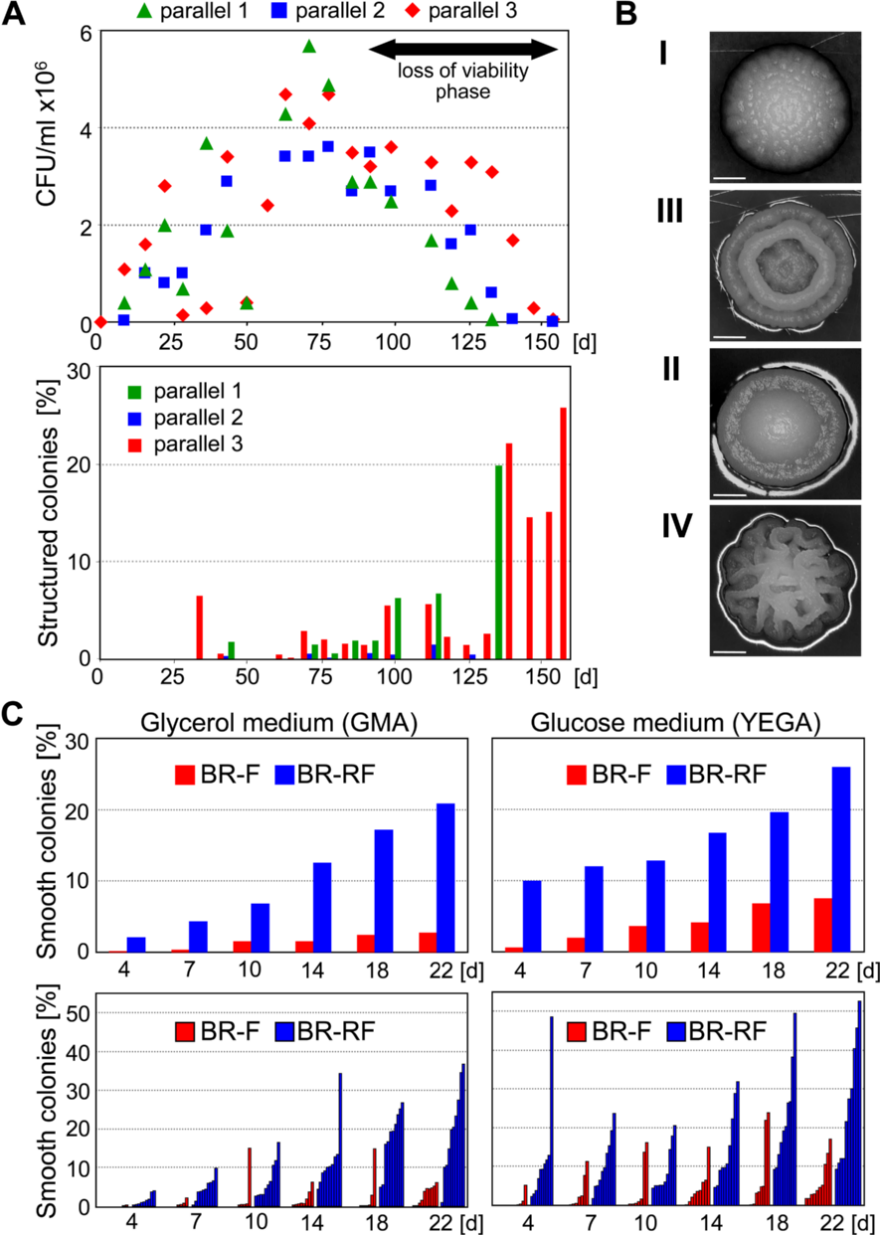
**Origin and stability of the BR-RF strain. A.** Upper panel, growth curve of 3 parallel static cultures of the BR-S strain in MM-2% ethanol medium, expressed as CFU/ml. Lower panel, appearance of cells forming structured colonies (switched phenotype) expressed as a percentage of total colony number. A representative experiment of the three is shown. **B.** Colonies with structured morphology originating from the BR-S strain during cultivation in static culture. From the top, morphotypes I, II, III and IV (the latter named BR-RF). Scale bar = 1 mm. **C.** Domestication frequency of colonies of the BR-F and BR-RF strains. The upper graphs represent the average appearance of smooth colonies after the re-plating of BR-F (red columns) and BR-RF (blue columns) colonies relative to the total CFU of all re-plated colonies. The bottom graphs show the switching frequencies of individual colonies, BR-F (red columns) and BR-RF (blue columns).

To induce the conversion of a BR-S strain to a strain with a wild-type phenotype, the opposite process of domestication, we set up various stressful and long-term starvation conditions. We incubated the BR-S strain statically (i.e., without shaking) for several months in various media with a limited carbon source. During incubation in MM medium with 2% ethanol, the number of colony-forming units (CFU) in the suspension was monitored. In parallel, the morphology (structured versus smooth) of the arising microcolonies was determined on GMA plates (Figure 1A). After inoculation, the static cell culture grew slowly until approximately the 60th day, as indicated by the slowly increasing number of CFU. From approximately the 75th day, the number of CFU started to decrease, suggesting a gradual dying of part of the population (Figure 1A). During this period, the number of cells forming smooth colonies decreased to a rate of about 5 × 10^4^ cells per ml of culture per day. Colonies with a structured morphology started to appear rarely among the smooth colonies on the 28th day or later. The frequency of their appearance reached approximately 0-7% of the CFU (Figure 1A). Interestingly, between days 30 and 110, the number of cells forming structured colonies increased to a rate of up to 400 to 2000 cells per ml of the culture per day in parallel cultures. Thus, at later stages of static cultivation (140 days), the proportion of structured colonies increased to as high as 26% (Figure 1A). In some static cultivations the cells forming structured colonies reached as high as 40% of all CFU (not shown). Structured colonies also emerged in static cultivations with MM medium that contained lower or higher ethanol concentrations. In addition, colonies appeared in cultivations without any carbon source, in which ethanol was replaced with NaNO_3_. When ethanol was replaced with fructose, structured colonies only appeared when the starting fructose concentration was 0.5% or lower; with a fructose concentration of 1 - 2%, no structured colonies were observed in cultivations lasting more than 200 days.

The morphology of the emerging structured colonies was not uniform, varying from moderately structured to a fully structured colony phenotype. We classified the emerging colonies into four typical colony phenotype categories (Figure 1B). Most of the isolated subclones were unstable; after re-plating, these colonies domesticated at a high frequency, thus preventing a more detailed characterization. The most stable subclones formed colonies of the IV^th^ category, phenotypically resembling biofilm colonies of the original wild BR-F strain. One of these subclones, designated BR-RF, was used for further analyses.

### The BR-RF strain domesticates more efficiently than the original wild BR-F strain

With the aim of monitoring the stability of the BR-RF strain, we compared the frequency of its domestication on fermentative and respiratory media (evaluated as the frequency of formation of smooth colonies) with that of the BR-F strain (Figure 1C). In BR-F colonies, the percentage of domesticated subclones ranged from 0.05% (4-day-old colonies) to 2.6% (22-day-old colonies) when grown on respiratory medium and from 0.6% (4-day-old colonies) to 7.5% (22-day-old colonies) when grown on fermentable medium (Figure 1C, upper graphs). In individual BR-F colonies, the frequency of domesticated subclones varied from 0% to 15% on respiratory and from 0% to 24% on fermentable medium (Figure 1C, bottom graphs). In BR-RF colonies grown on respiratory GMA, domesticated subclones appeared with an average frequency of 2% (4-day-old colonies) to 21% (22-day-old colonies), with variation in individual colonies from 0% to 37%. Similar to BR-F domestication, the frequency of BR-RF domestication was even higher when the BR-RF colonies were grown on fermentable YEGA; domestication varied from 10% (4-day-old colonies) to 26% (22-day-old colonies), with variation in individual colonies from 2% to 53% (Figure 1C).

### Morphology and physiology of colonies formed by the BR-RF strain

**Figure 2:**
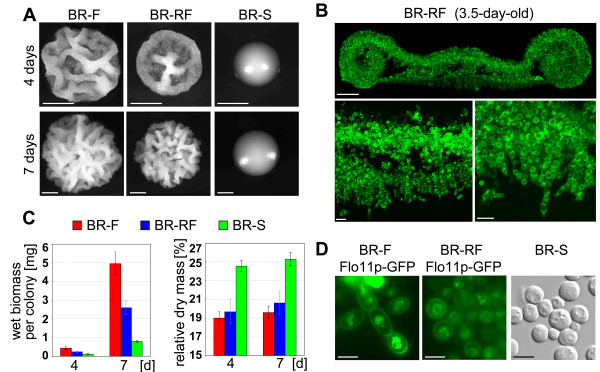
**Ultrastructure and physiological properties of colonies of the BR-RF strain and its ancestors. A.** Colonies of *S. cerevisiae* BR-F, BR-RF and BR-S strains. Scale bar = 1 mm. **B.** Vertical transversal cross-section of an 3.5-day-old BR-RF colony stained with ConA-AF and visualized by 2P-CM. Scale bar = 100 μm. At the bottom, magnification of the central region of the BR-RF colony (left) and lower subsurface part of the colony (right). Scale bar = 10 μm. **C.** Physiological characteristics of BR-F, BR-RF and BR-S colonies. Left, amount of wet biomass per colony. Right, relative amount of dry mass. **D.** Localization of Flo11p-GFP and morphology of cells taken from 4-day-old BR-F and BR-RF colonies. Right, Nomarski contrast image of cells from 4-day-old BR-S colonies. The Flo11p-GFP production in these colonies was below the detection level. Scale bar = 5 μm.

The morphology of colonies (bird’s-eye view) formed by the BR-RF strain was identical to that of colonies formed by the original BR-F strain (Figure 2A). Two-photon excitation confocal microscopy (2P-CM) confirmed that the BR-RF colony was composed of an aerial part and of subsurface roots attaching the colony to the substrate. This composition is similar to the organization of the BR-F colony [16]. The colonies of both strains contained internal cell-free cavities (Figure 2B). In contrast to BR-F colonies [16], BR-RF colonies were formed exclusively of oval cells, including the subsurface parts where chains of oval cells invaded the agar (Figure 2B) instead of the pseudohyphae that are typical of BR-F colonies. Thus, although the BR-RF strain gained the ability to form fully structured colonies, it did not revert its cell morphology and retained the oval cells typical of the BR-S strain (Figure 2D).

Although they formed similarly structured colonies, BR-F and BR-RF strains may have differed in other physiological parameters. We therefore analyzed BR-RF colonies grown on GMA for prominent characteristics previously found to be different between colonies formed by the BR-F and BR-S strains [14]. The analysis revealed that in some aspects, BR-RF colonies behaved as an “intermediate” between BR-F and BR-S colonies. This intermediate behavior was mostly related to wet biomass accrual (Figure 2C) and colony size (Figure 2A), while the water content (relative amount of dry mass) of BR-RF colonies was almost identical to BR-F colonies (Figure 2C).

**Figure 3:**
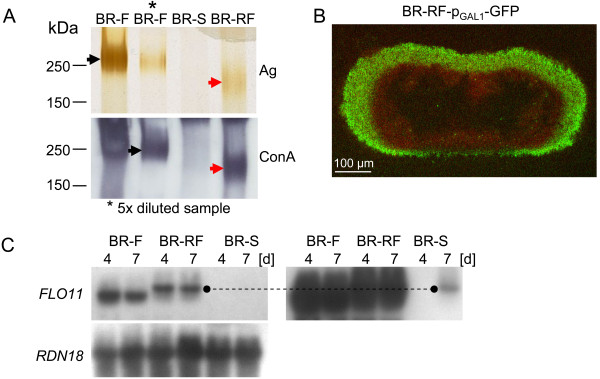
**Visualization of the ECM, presence of HMWGP protein and *FLO11 *mRNA in BR-RF colonies. A.** Glycoproteins in extracts from BR-F, BR-S and BR-RF colonies stained with silver (Ag) and visualized by the ConA/peroxidase (ConA) method; arrows indicate HMWGP in BR-F (black arrows) and in BR-RF (red arrows) colonies. **B.** Visualization of the ECM in vertical cross-sections of BR-RF-p_GAL1_-GFP colonies using 2P-CM. Green, GFP fluorescence indicates areas in which the inducer (galactose) reached the cells; red, autofluorescence of all colony cells visible in areas where the ECM prevented the inducer from accessing the cells. Intact colonies were induced from the bottom by placing them for 5 h on agar soaked with 2% galactose [16]. **C**. *FLO11* mRNA in 4- and 7-day-old BR-F, BR-RF and BR-S colonies. Right, stronger exposure revealed low *FLO11* mRNA levels in BR-S colonies. The discontinuous line compares the length of the *FLO11* transcripts in BR-RF and BR-S cells. The *RDN18* gene was used as an RNA loading control.

Another typical feature of BR-F colonies was the presence of high-molecular-weight glycosylated protein (HMWGP), a possible constituent of the extracellular matrix (ECM). The production of HMWGP was switched off in BR-S colonies [13]. BR-RF colonies restored production of this protein, but at a lower level than in BR-F colonies. In addition, SDS-PAGE showed that the mobility of this protein was shifted to lower MW. This observation suggested that the molecule was shortened or underwent less glycosylation (Figure 3A). The presence of HMWGP (an ECM constituent) and a high content of water, possibly trapped by ECM [14] implied that BR-RF colonies restored ECM production. To demonstrate presence of ECM we prepared a BR-RF-p_GAL1_-GFP strain to monitor the ECM in the developing colony by 2P-CM, as previously described [16]. As in BR-F colonies [16], internal BR-RF colony regions were also protected via a low-permeable ECM (Figure 3B).

The production of the adhesin Flo11p is indispensable for the formation of structured colonies [14,17], and Flo11p production is switched off during wild strain domestication [13]. Both *FLO11* gene expression (Figure 3C) and Flo11p protein production (Figure 2D) are recovered in the BR-RF strain. Interestingly, the *FLO11* mRNA of the BR-RF strain is longer than that of the BR-F strain (Figure 3C).

### Genome-wide expression differences between BR-F, BR-RF and BR-S colonies

**Figure 4:**
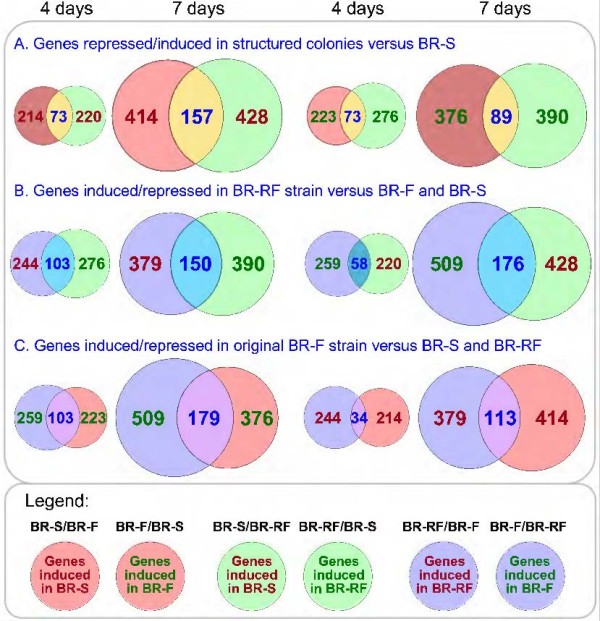
**Comparison of transcriptomes from cells forming BR-F, BR-RF and BR-S colonies.** Venn diagrams comparing genes induced in particular strain comparisons as indicated in titles of **A**, **B** and **C** panels. Red circles, comparison of BR-F versus BR-S; green circles, comparison of BR-S versus BR-RF; and blue circles, comparison of BR-F and BR-RF (see also the legend at the bottom of the figure).

To obtain an overall view of the transcription characteristics typical of the biofilm colony phenotype, we isolated total RNA from 4-day-old and 7-day-old colonies formed by the BR-F, BR-S and BR-RF strains. We then compared the transcriptomes of the BR-RF versus BR-F strain, the BR-RF versus BR-S strain and the BR-F versus BR-S strain using a microarray (Additional file 1: Table S1). Mutual comparison of the transcribed genes enabled the identification of genes specifically activated or repressed in complex structured biofilm colonies (represented by the BR-F and BR-RF strains) compared to smooth colonies (represented by the BR-S strain) (Table 1). However, the expression differences related to the structured biofilm colony phenotype were only a subset of the expression differences observed between the strains. Other extensive differences unrelated to the colony phenotype were observed between BR-RF and BR-F colonies, as well as between BR-RF and BR-S colonies. Some of these differences are summarized in Figure 4 and discussed below. This finding supports the data described above indicating that some properties of BR-RF colonies differ from colonies formed by both BR-F and BR-S.

**Table 1:** Functional classification of genes up-regulated in particular colony phenotypes

	**Structured biofilm colony morphotype**	**Smooth colony morphotype**
**Cell wall**	SVS1, **ASP3-1,-2,-3,-4, BSC1,***OSW5*	**SPS100, CTS1** ***,*** *FLO9, PFS1, PSA1, ZPS1*
**Secretory transport & processing**	BST1, LST7, PER1, **CWH41, MNN9,***YOL107W, APM4, GVP36, YIP4*	BTN2, NCE102, **GYP7***, KTR4, PEP12, SNC2, TIM17, TIM21*
**Metabolism**	JLP1, MLS1, SNZ1, SNZ2, TPI1 **—***YMR226C, BNA1, ENO1, HPT1, MDH2, RHR2, SOL4*	DSF1, PIG2, **STR3**, *CRS5, CUP1-1, FBP26, FOL2, FRE6, HXK1, MTD1, NFS1, SFA1*
*Amino acids*	ARG3, ARG4, BAT1, MET17, MET22, **ARG1,***GDH3, LAP3*	*GCV1, GCV2, GCV3, SHM2*
*Lipids & fatty acids*	INO1 **—***ECI1, POT1, POX1, TES1, VPS66*	YNL144C, ERG3, ERG4, HFD1 — *AUR1*, *CEM1*, *CSG2, SUR1, SUR4*
*Nucleotides*		RNR4 **—***ADE1, ADE2, ADE8, ADE13, PRS3*
**Transport**	ITR1, PUT4, VHT1, **PDR12, VBA3,***ATO2, CAN1, GAP1, MEP2, PIC2*	YFL054C HXT15, MUP3, PHO84, THI74, ZRC1, **HXT5**, **HXT17**, **PHO89**, *YFL054C, HXT1, HXT2, HXT3, HXT4, HXT6, HXT10, , HXT12, HXT13, MAL11*, *PHO87, ZRT2*
**Degradation**	*HSP33, MGR1, NAS2, YSP3*	*ATG8, BSD2, CIC1, DFG16, GID8, JID1, UMP1, RPN5, RPN13, RPT2, SNA3, VID24, YPT53*
**Stress response**	DDR48, GRX3 **—***TSA1, YBL036C, CTA1, SOD2, TSA2*	OLA1, HSP42, SPG1, SSA3, SSA4, **APJ1, FES1**, **GPX1, HSP30, HSP78, SSE1,***YNL190W, AAD6, CCT5, CPR6, GLO1, GSH1, HSP104***,***HSP150, MGE1, OYE3, PST1, RCN2, SED1, STI1, TRX3, YDJ1,YGP1*
**Regulation**		
*Translation & RNA processing*	BRR2, MTQ2, SLF1, **DUS4**, **SPP381**, *IST3, MRS2, RPP1A, RPR2*	PUS2, SLH1**, NGL3, RPA135**, **RSC30,***YDR341C, CBF5, SRO9, RPP2A, RPS30B, RPL35B, RRP3, RRP7, SRC1*
*Signal transduction*	*FAR3*	GPA2, MTH1, MRK1, STD1, **LST8**, **RGS2,***YMR291W, GLC8, HRK1, KIC1, MTH1, STE7, YAK1*
*Cell cycle & polarity*	**SHE1**	PCL9, HBT1, TAH11, **CLG1,***CDC13, CDC3, DSE1, VHS1*
*Transcription*	CUP9, SFG1, TEC1, **YAP7,***DAL80*, *RDS1*	**YFL052W, HMS1**, **RLM1, USV1**, **ZPR1**, *ALPHA2, CIN5, GIS1, HAP4, OPI1, PHO2, STP2, STP4*
*- chromatin structure*	HHF1	**TOS8***, IOC4, HHT1, TMA23,TOD6*
**General transcription**		*IWR1, RBA50, RPA135, SPT15, SUA7, TAF12, TFA1*
Transposons*	8 genes, **10 genes,** 1 *genes*	
Unknown*	7 genes, **5 genes,***17 genes*	8 genes, **4 genes,***11 genes*
**Others**		YLR164W, **DIA1, DIA3, MRH1, MRPS17,***IXR1, PIN3*

Genes only expressed differently on the 4th day are in standard letters, genes differently expressed on both the 4th and 7th day are in bold, and genes only expressed differently on the 7th day are italicized.

*genes are listed in the Additional file 1: Table S1 and discussed in the text.

### Expression characteristics of complex biofilm colonies

Comparison of the structured biofilm colony versus the smooth colony transcriptomes revealed 73 and 89 genes with significantly higher expression in 4- and 7-day-old structured colonies, respectively. In smooth colonies, the expression levels of 73 and 157 genes were increased in 4- and 7-day-old colonies, respectively (Figure 4, Table 1, Additional file 1: Table S1).

The structured biofilm colonies up-regulated genes involved in protein secretion and modification. Some of these genes, such as *MNN9*, *CWH41*, *BST1* and *PER1,* influence the structure and the composition of the cell wall and/or the membrane. In addition, the structured colonies highly up-regulated genes for cell wall proteins such as *BSC1*, which is similar to the *FLO11,* and *ASP3* genes. On the other hand, the structured colonies down-regulated the expression of other cell wall-related genes such as *SPS100* and *CTS1* as well as genes related to secretion and protein modification. These data indicate that structured and smooth colonies differ in the carbohydrate and protein composition of their cell wall (Table 1).

The structured and smooth colonies also differed in their expression of metabolic genes and nutrient transporters. The structured colonies up-regulated amino acid metabolism genes and transporter genes, including genes involved in arginine and methionine metabolism. The structured colonies also expressed genes involved in phospholipid biosynthesis and fatty acid β-oxidation, and they expressed the inositol transporter gene *ITR1*. In contrast, the few amino acid metabolism genes that were up-regulated in the smooth colonies were for the mitochondrial glycine decarboxylase complex. The smooth colonies also up-regulated a number of nucleotide metabolism genes, genes involved in copper and iron resistance and homeostasis and several genes related to lipid metabolism. These included genes involved in ergosterol, sphingolipid and ceramide metabolism (*ERG3*, *SUR1*, *SUR4*, *CSG2*). The transporter genes included those for the transport of hexoses and phosphate (*PHO84*, *PHO87*) (Table 1).

In addition, the smooth colonies up-regulated a large group of genes related to the stress response (28 genes), including genes for various chaperones, genes involved in cell wall stress response/maintenance and certain genes involved in the oxidative stress response. The few stress response genes induced in structured colonies included oxidative stress response genes (*GRX3*, *TSA1*, *TSA2*, *CTA1* and *SOD2*) and genes involved in DNA damage response and repair (*DDR48*, *YBL036C*, *DDI3* and *RAD10*) (Table 1).

The cells of structured and smooth colonies also differed in their expression of a remarkable number of genes involved in signaling cascades (1 gene up-regulated in the structured and 13 genes in the smooth colonies), transcription regulators (6 genes up-regulated in the structured and 13 genes in the smooth colonies) and genes involved in translation and RNA processing (9 genes up-regulated in the structured and 14 genes in the smooth colonies). The transcription factor genes up-regulated in the structured colonies included *TEC1* and *SFG1*, which are involved in yeast filamentation and pseudohyphal growth. In contrast, the smooth colonies up-regulated *RLM1*, *YFL052W* and *USV1*, which are related to cell wall integrity. They also up-regulated several genes related to chromatin structure and remodeling (*TOS8*, *TOD6*, *TMA23*, *IOC4* and *HHT1*) (Table 1).

In addition, the structured colonies predominantly expressed genes for transposons and a large group of genes with unknown function, some of which were localized to the subtelomeric regions (Table 1).

### BR-RF colonies up-regulated expression of genes located at specific positions in chromosomes

BR-RF colonies increased the expression of 244 genes and 379 genes in 4- and 7-day-old colonies, respectively, compared with BR-F colonies. BR-RF colonies reduced the expression of 259 genes and 509 genes in the 4- and 7-day-old colonies, respectively, compared to BR-F colonies. For the comparison between the BR-RF and BR-S strains, BR-RF colonies increased the expression of 276 genes and 390 genes in 4- and 7-day-old colonies, respectively, compared to BR-S colonies (including typical “biofilm colony” genes, see above). BR-RF colonies repressed 220 and 428 genes in 4- and 7-day-old colonies, respectively, compared to BR-S colonies. In addition, a relatively large number of genes were up-regulated (103 genes in 4-day-old and 150 genes in 7-day-old colonies) and down-regulated (58 genes in 4-day-old and 176 genes in 7-day-old colonies) in BR-RF colonies when compared with both BR-F and BR-S colonies (Figure 4).

**Figure 5:**
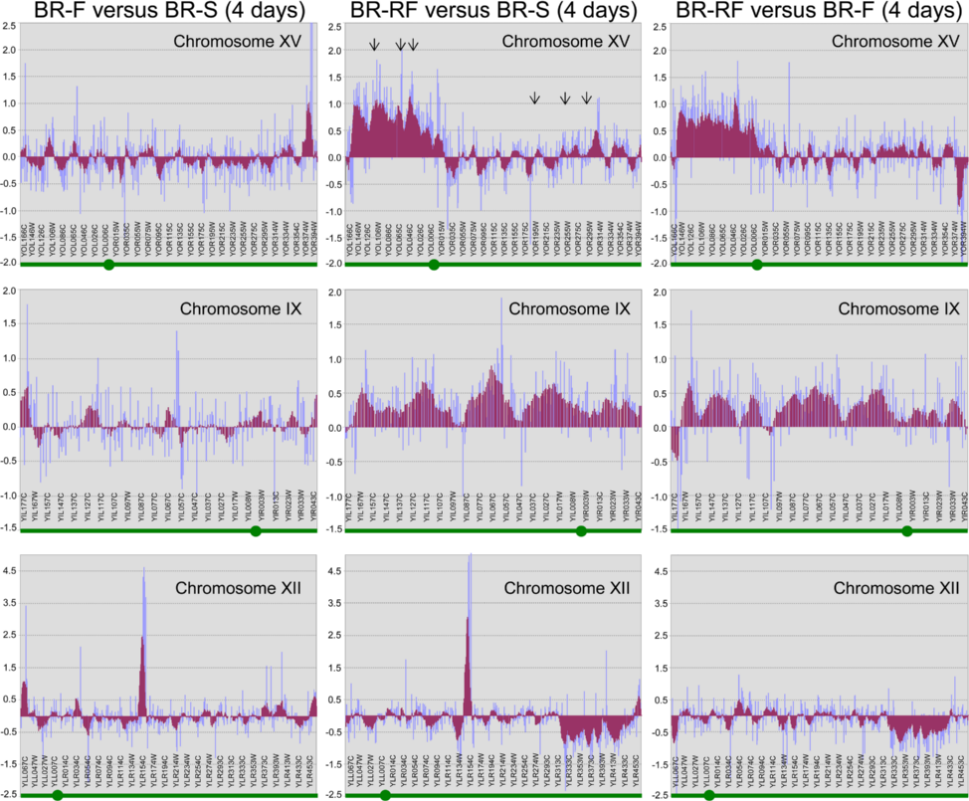
**Position clustering of differently expressed genes on chromosomes XV, IX and VII.** Positional gene enrichment analysis performed on the basis of expression comparison of 4-day-old colonies of BR-F versus BR-S, BR-RF versus BR-S and BR-RF versus BR-F. X axis, position of each gene along the particular chromosome, the position of the centromere is marked by a green circle on green bar on the bottom of each graph; Y axis, relative expression of a particular gene. Blue columns, experimental expression values (expression ratios in log_2_); red columns, simple moving averages of experimental log_2_ values of 10 neighboring genes. Black arrows indicate position of ORFs analysed by qPCR as shown in Figure 6.

“Positional Gene Enrichment” analysis revealed that many gene expression differences between the BR-RF strain and either the BR-F or BR-S strain (or both) involved the expression of genes localized to specific chromosomal regions (Figure 5) rather than genes randomly distributed in the genome. Most prominently, a high number of genes up-regulated in 4-day old colonies of BR-RF strain were localized to a 300-kbp region of the left arm of chromosome XV. This region included 33% of all activated genes in the BR-RF versus BR-S strain, 32% of all activated genes in the BR-RF versus BR-F strain and 60% of all activated genes in the BR-RF versus both BR-S and BR-F strains. Smaller groups of genes up-regulated in the BR-RF strain versus either the BR-S or BR-F strains were localized to a 320-kbp region of chromosome IX , and a group of BR-RF strain genes that were down-regulated compared to the BR-F and BR-S strains was localized to a 240-kbp region of chromosome XII (Figure 5). In contrast, neither the gene expression differences between the BR-F and BR-S strains, nor the genes expressed specifically in structured biofilm colonies (i.e., those differently expressed between the BR-F and BR-RF strains versus the BR-S strain) were localized to specific chromosomal regions (not shown).

**Figure 6:**
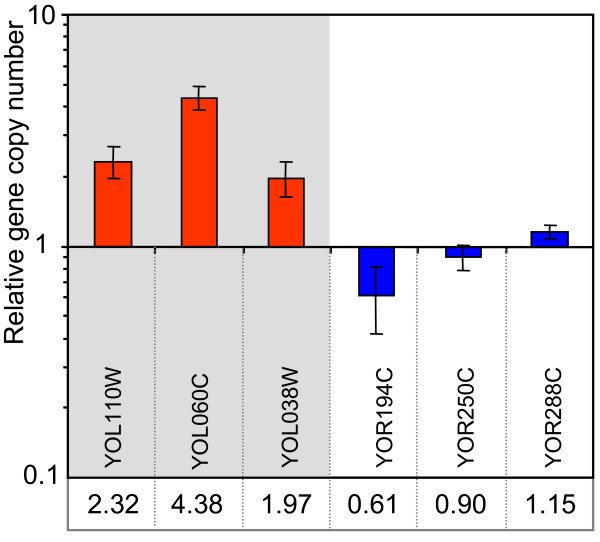
**Relative copy number of genes located on the left and right arm of chromosome XV in BR-RF versus BR-S.** Comparison of relative copy number was performed by qPCR using chromosomal DNA isolated from BR-RF and BR-S strains. Positions of examined genes on chromosome XV are marked by black arrows at Figure 5. Red, genes located at the left arm of the chromosome XV the expression of which is upregulated in BR-RF when compared with BR-S (and BR-F); blue, genes located at the right arm of the chromosome XV. Y axis, relative gene copy number (BR-RF versus BR-S); the exact values are stated below the ORFs. The mean of values from 3 independent experiments ± SD are shown; data significance (relative copy number of each left-arm-gene compared to relative copy number of each right-arm-gene) was determined using the two-tailed *t* test. All P values varied between P < 0.05 and P < 0.005.

As the higher level of gene expression related to distinct chromosomal areas could be caused by DNA duplications, we measured the copy numbers of 3 genes located in the left arm of chromosome XV (the genome region with higher expression in BR-RF), relative to those of 3 genes located in the right arm of the same chromosome using real time qPCR. The results (Figure 6) showed significant differences between gene copy numbers in the left and right arms of chromosome XV in BR-RF indicating partial genome duplications.

### BR-F colonies up-regulate expression of genes located in HAST domains, which affect BR-F stress resistance

Analysis of the differently expressed genes revealed that some are localized near telomeres. It has been hypothesized that transitions between different phenotypes are controlled by transcriptional silencing via specific chromatin-remodeling enzymes [18,19]. Thus, we compared the genes that were expressed differently in the BR-F, BR-S and BR-RF colonies with the list of genes expressed differently in a mutant containing a deletion of *SIR2*[20], the gene encoding histone deacetylase. Histone deacetylase Sir2p has been shown to affect transcription at specific chromosomal locations near telomeres [21]. This analysis revealed a correlation between the genes down-regulated in the *sir2Δ *strain and the genes typically expressed in colonies with a biofilm phenotype (29% of genes up-regulated in 4-day-old structured biofilm colonies). However, neither the structured colony morphology nor the domestication rate of the BR-F-*sir2* strain (in which both *SIR2* alleles were deleted) was changed when compared with the BR-F strain (data not shown). This result implies that the function of Sir2p is not important for the formation of structured colony morphology.

Next, we compared our set of genes with a database of genes specified to be under the control of another deacetylase, Hda1p. Hda1p was shown to cause transcriptional repression in specifically localized subtelomeric chromosomal regions [22]. This analysis revealed a correlation between the genes localized to HAST domains, i.e., regions controlled by Hda1p histone deacetylase [22], and the genes up-regulated in BR-F colonies. In particular, 18% of the genes up-regulated in BR-F versus BR-S colonies, 19% of genes up-regulated in BR-F versus BR-RF colonies and 32% of genes up-regulated in BR-F versus both BR-RF and BR-S 4-day-old colonies were localized to these domains. In contrast, upregulation of HAST domain genes in BR-S and BR-RF colonies versus BR-F colonies was less than 6%. Thus, the upregulation of these genes in BR-F colonies indicated decreased activity of Hda1p compared to BR-S and BR-RF colonies.

**Figure 7:**
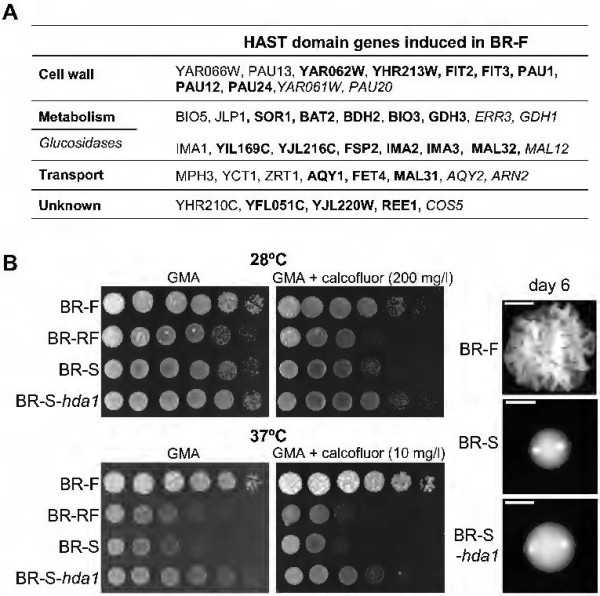
**Derepression of HAST domain genes. A.** Genes localized to HAST domains [22] up-regulated specifically in 4- and 7-day-old BR-F colonies compared to both BR-RF and BR-S colonies. Genes only expressed differently on the 4th day are in standard letters, genes expressed differently on both the 4th and 7th day are in bold and genes only expressed differently on the 7th day are italicized. **B**. Left, drop assays of cells from BR-F, BR-RF, BR-S and BR-S-*hda1* colonies plated on GMA supplemented with calcofluor white and grown at 28°C and 37°C as indicated. Right, 6-day-old colonies of BR-F, BR-S and BR-S-*hda1* strains. Scale bar = 1 mm.

Genes in the HAST domains that were specifically up-regulated in the BR-F strain (Figure 7A) included genes encoding various glucosidases (8 genes), proteins with mostly unknown function and predicted localization to the cell wall (11 genes) and various transporters (8 genes). These data indicated that the cells in the BR-F colonies could have specific cell wall and plasma membrane properties compared to the BR-S and BR-RF colonies; however, these properties seemed unrelated to the structured biofilm colony phenotype. To test this prediction, we prepared the strain BR-S-*hda1,* with both alleles of the *HDA1* gene deleted, which should de-repress HAST domain genes. We then analyzed the morphology of colonies of this strain and the resistance to various stresses compared with the BR-F, BR-RF and BR-S strains (Figure 7B). The BR-S-*hda1* strain formed smooth colonies similar in morphology but slightly larger than colonies of the BR-S strain of the same age (Figure 7B). Similarly to the BR-F strain, the BR-S-*hda1* strain was significantly more resistant to the chitin binding dye calcofluor white, and it grew better at a higher temperature (37°C) than either the BR-S or the BR-RF strain. Thus, deletion of the *HDA1* gene in the BR-S strain increased its calcofluor white and temperature resistance to the same level as the BR-F strain, but it did not change the smooth colony morphology.

## Discussion

Wild *S. cerevisiae* strains are able to effectively domesticate when transferred to favorable laboratory conditions [13,14]. Here, we show that the BR-S strain can switch back to a wild-type-like phenotype and start to form structured biofilm colonies following long-term exposure to starvation and stress conditions. In contrast to the original BR-F strain, which forms structured colonies with a uniform morphology, our isolated feral BR-S subclones form colonies with different phenotypes. Thus, the increased phenotypic variability of the feral BR-S subclones seems to be induced by environmental stress and starvation. These data agree with previous findings that non-isogenic *S. cerevisiae* strains isolated from various habitats can form differently structured colonies [14], similarly to pathogenic fungi [5].

The first feral subclones of the BR-S strain appear after approximately 30 days of slow growth in static cultures with limited nutrients. Later, the proportion of subclones increases up to 26% of the viable cells in some of the parallel cultivations. At least between days 75 and 110, such an increase could be caused by an increased rate of phenotypic switching as well as by the preferential dying of smooth cells during the “loss of viability” phase of the population growth curve (Figure 1A). The number of feral cells starts to decrease from day 115, i.e. 40 days later that in the case of domesticated cells. These findings suggest that feral subclones gain the ability to better survive starvation than domesticated cells. Higher concentrations of a better carbon source (e.g., fructose) block the emergence of subclones forming structured colonies, thus indicating the importance of nutrient restriction for the evolution of feral subclones. Similarly, various stress factors promote phenotypic switching of *C. albicans*, resulting in the production of cell variants that are more resistant to stresses [23]. Exposure of *C. neoformans* cells to various host defense mechanisms in vivo also leads to phenotypic modulation of an originally homogeneous population and to the subsequent prevalence of variants that are more resistant to the host immune response [6].

A BR-RF strain forms structured colonies with a morphology that is visually identical to that of the colonies formed by the original wild BR-F strain. The BR-RF strain, however, differs in several aspects from the BR-F strain. First, the BR-RF strain domesticates 4 times more effectively on fermentable media and 8 times more effectively on respiratory media than the BR-F strain. This finding suggests that the several months of starvation are sufficient to induce the formation of feral subclones but are not sufficient to make the feral subclones as stable as the original BR-F strain, which evolved over a much longer time in a natural setting. This finding also agrees with the results from some feral subclones that emerged during static cultivation (Figure 1B); these subclones form less structured colony phenotypes that are even more unstable and quickly revert back to the domesticated phenotype. The data also demonstrate that the presence of a good fermentable carbon source such as glucose efficiently promotes the domestication of both strains (Figure 1C) and prevents the switch to a structured phenotype as indicated above.

Second, the colonies of the BR-RF strain differ from the colonies of both the BR-F and BR-S strains in certain physiological parameters, behaving as an “intermediate” between the BR-F and BR-S colonies (Figure 2). Similarly to the BR-S strain, the BR-RF strain also forms oval cells and is not able to form the elongated cells and pseudohyphae that are typical of the BR-F strain. This finding supports the idea that the particular cell morphology is a dispensable factor in the formation of biofilm colony architecture [14].

On the other hand, both the formation of the ECM and the production of Flo11p adhesin, the traits important for the formation of structured colony morphology [14], recover in BR-RF colonies. The length of the *FLO11* transcript, however, differs from the original BR-F and the feral BR-RF strains. Recombination of the central repeat domain of the yeast adhesin genes is frequently observed [24], which often leads to the generation of a protein with different and/or enhanced adhesive function [25]. The change in the *FLO11* gene of the BR-F strain most likely occurs during the domestication process; the *FLO11* transcript, although present at very low levels in BR-S colonies, is the same size in both the BR-S and BR-RF strains. Thus, it seems that rather than actual gene size and potentially protein size, the Flo11p expression level is important in the context of structured colony formation. The altered *FLO11* transcript length also indicates that DNA recombination may occur frequently during phenotypic transitions of the BR-F strain. This conclusion is further supported by the observed differences in the MW of the HMWGP protein component of the ECM (Figure 3), which could be caused by different posttranslational modifications or by changes in gene structure. In summary, these data show that the BR-RF strain, which was selected under the conditions of long-term nutrient deprivation and stress, develops some properties similar to the BR-F strain (e.g., the formation of structured biofilm colony morphology) but also maintains some properties of the BR-S strain (e.g., the formation of oval cells).

Genome-wide transcriptome analysis allows us to determine the typical gene expression profile for a structured colony phenotype compared with the smooth phenotype. Structured biofilm colonies (BR-F and BR-RF) both up-regulate and down-regulate distinct genes involved in cell wall remodeling as well as genes involved in the delivery and modification of secreted and cell wall/membrane-attached proteins. This finding indicates that, in addition to Flo11p adhesin, the carbohydrate composition of the cell wall, the appearance of specific surface proteins and protein glycosylation may affect specific properties of the cells (such as the production of the ECM) and contribute to the formation of a biofilm colony. Ty transposon genes and several unknown genes that are expressed in biofilm colonies are located in subtelomeric regions. This suggests that transcription of genomic regions that are influenced by chromatin structure [26] differs in biofilm colonies compared to smooth colonies. In support, smooth colonies up-regulate the expression of a group of genes involved in histone modification and chromatin remodeling (Table 1).

There is further evidence of the importance of chromatin remodeling in the diversification of properties of phenotypic variants derived from the BR-F strain. BR-F colonies (but not BR-RF colonies) express genes located in HAST domains, i.e., regions in the genome whose expression is modulated by histone deacetylase Hda1p [22]. Deletion of the *HDA1* gene (and thus de-repression of HAST domain genes) significantly increases the resistance of BR-S cells to both calcofluor white and high temperature, thereby reaching the level of resistance of the BR-F strain. Calcofluor white interferes with cell wall assembly [27], and strains with mutations that affect the cell wall composition are more sensitive to calcofluor white [28]. Thus, the resistance of the BR-F strain could be caused by expression of HAST domain genes encoding various cell wall and plasma membrane-related proteins. In support of persistent repression of HAST domain genes in BR-RF colonies, cells from BR-RF colonies behave similarly to BR-S cells. On the other hand, de-repression of HAST domain genes in BR-S-*hda1* colonies does not affect the smooth morphology of these colonies, indicating that HAST domain genes expressed in the BR-F strain are unrelated to the biofilm colony phenotype. However, other studies demonstrate the role of specific deacetylases, including Hda1p, in controlling the phenotypic transition of the “white-opaque” *C. albicans* colony [18,29]. Hence, epigenetic mechanisms mediated by chromatin remodeling enzymes have the capacity to regulate phenotypic heterogeneity.

In addition to the expression characteristics specific to structured biofilm colonies, gene expression specific to BR-RF colonies is related to three regions of the BR-RF genome. This finding indicates that some chromosome rearrangements occur during BR-RF strain formation; these could result in the duplication of certain parts of chromosomes or in chromosomal rearrangement(s) relocating the regions to more actively transcribed locations of the genome. Real time qPCR comparison of relative copy number of three genes located in the left arm of chromosome XV that upregulate expression in BR-RF (*YOL110W*, *YOL060C* and *YOL038W*) with that of three genes located in the right arm of the same chromosome (*YOR194C*, *YOR250C*, *YOR288C*), indicates large duplications in the genome (Figure 6). This finding also agrees with previous observations that diverse conditions can evoke aneuploidy in yeast, which can directly impact gene expression, as detected at both the transcriptional and proteomic levels [30,31], and can also modulate the switch between different colony phenotypes [32]. However, the observed chromosome site-specific expression characteristics of the BR-RF strain are unrelated to the structured colony phenotype, as only a few biofilm colony-specific genes are localized to these chromosomal regions. DNA rearrangement events leading to karyotypic instability are also observed during phenotypic transitions of certain *C. albicans*[33] and *C. neoformans* strains [8]. These previously observed changes are not correlated with phenotypic variability and are irreversible. Thus, it seems that DNA rearrangements resulting in particular gene expression changes are not the cause of the BR-F/BR-S/BR-RF phenotypic switch, which is in contrast to recent findings in other wild strains [32]. Instead, these rearrangements in the BR-RF strain could be a side-product of the increased rate of recombination events that occur during the phenotype transitions.

## Conclusions

We describe the phenotypic, genomic and gene expression differences among three *S. cerevisiae* strains (wild, domesticated and feral) that emerged by phenotypic switching under diverse environmental conditions. These strains form colonies with varying complexity and gain diverse features including altered resistance to stress. We show that the wide variability of natural *S. cerevisiae* strains can be further potentiated under stressful environmental conditions. We identify genes specifically expressed in the structured colony phenotype, such as genes that specifically affect the composition of cell surface structures; these genes provide clues as to the specific processes involved in the formation of structured colony architecture. We also document a role of Hda1p histone deacetylase in strain resistance to stress. In addition, our data indicate that genomic rearrangement(s) occur in the feral strain that are unrelated to the phenotypic switch. These changes contribute to expression characteristics of the feral strain that are distinct from both its ancestors, the wild and domesticated strains.

In summary, our findings show that extensive phenotypic modulation occurs in wild *S. cerevisiae* strains. Phenotypic switching can be controlled by various mechanisms and enables flexible adaptation of these strains to a particular environment in the wild. The comparison of the three originally isogenic strains BR-F, BR-S and BR-RF revealed many significant gene expression and phenotypic differences, but only a few of them seem to be related to the formation of structured biofilm colonies. In addition, the differences observed between BR-F and BR-RF showed that reverted structured colony morphology does not necessarily mean reversion of other strain properties. In other words, strains with different properties such as BR-F and BR-RF can form structured colonies. Thus, an important question remains as to whether there is a single dominant mechanism that forces yeast strains to form structured colonies independently of other properties or, more probably, a variety of mechanisms that support the formation of structured colony morphology.

## Methods

### Strains and media

All strains and their derivatives are listed in Additional file 2: Table S2. The wild *S. cerevisiae* strain BR-F, originally collected from the lake in south Slovakia [34] is from the collection of the Chemical Institute of the Academy of Science, Bratislava, Slovak Republic (collection number CCY 21-4-97). The BR-RF and BR-S strains are isogenic to the BR-F strain and arose from phenotypic switching. Colonies were grown on GMA (3% glycerol, 1% yeast extract, 2% agar) or YEGA (2% glucose, 1% yeast extract, 2% agar) media at 28°C unless otherwise indicated. For static cultivations, we used liquid minimal medium (MM) (0.5% (NH_4_)_2_SO_4_, 0.1% KH_2_PO_4_, 0.05% MgSO_4_, pH 5–6.5) supplemented with ethanol (0.1%, 0.5%, 1%, 2% and 3% v/v), fructose (0.2%, 0.5%, 1% and 2%) or NaNO_3_ (0.01% w/v). For selective plates, media were supplemented with 200 mg/l G418 or 100 mg/l nourseothricin.

### Static cultivations, determination of CFU and percentage of cells that switched to form biofilm colonies

The BR-S strain was inoculated into MM medium at a concentration of approximately 5×10^5^ cells per ml. The cell culture was split into 10 ml portions in glass tubes (3 to 10 parallels for each particular concentration of the supplements) and statically cultivated at 28°C. During long-term cultivation, the culture was mixed at particular time-points. After appropriate dilution, 100 μl aliquots were plated on GMA to determine the number of CFU and the proportion of cells forming structured biofilm colonies.

### Construction of the strains

Flo11p-GFP strains containing the *FLO11* gene fused with the GFP gene were constructed via a previously described procedure [17]. The BR-RF strain with an artificial promoter p_GAL1_-GFP cassette integrated in the genome instead of the *HIS3* gene was constructed as previously described [16]. Gene knock-outs were performed by transforming the cells with deletion cassettes generated by PCR using the primers and plasmids listed in Additional file 2: Table S3. Yeast cells were transformed according to a published protocol [35].

### Determination of colony biomass

The biomass of colonies was estimated as their wet weight. To determine dry biomass, the wet biomass was dried in a Speed-Vac for at least 6 h. The water content was calculated as the difference between the wet and dry biomasses. The data were presented as the average of three independent experiments ± SD (standard deviation).

### Determination of switching frequency

The occurrence of switching from the BR-F and BR-RF to the BR-S colony phenotype was determined by visually scoring the colonies of different ages growing either on GMA or on YEGA media. To calculate the switching frequencies, 100–200 BR-F and BR-RF cells were plated on GMA plates, and at least 5000 colonies were scored. The percentage of colonies of the total CFU with smooth morphology was determined.

### Fluorescence microscopy of cells and colony imaging

Cells were examined under a Leica DMR microscope equipped with a 100×/1.3 oil objective and a GFP filter or Nomarski contrast and photographed with a ProgRes® MF^cool^ CCD camera (Jenoptik, Germany). Colony images were captured in incident light. A ProgRes® CT3 CMOS camera with a Navitar objective, Fiber-Lite PL-800 illumination system and NIS Elements software (Laboratory Imaging) were used.

### Sensitivity of the strains to calcofluor white

The strain sensitivity was tested by the drop assay. Four-day-old colonies were harvested to make a suspension containing 2×10^8^ cells per ml. Next, 5 μl drops of 10-fold serial dilutions were applied to GMA plates supplemented with 10 mg/l or 200 mg/l calcofluor white. Plates were scored after 3 days of incubation at 28°C or 37°C.

### Northern blot analysis

Total RNA from the cells of colonies grown on GMA plates was isolated by the hot phenol method as previously described [14]. For northern blots, 12 μg of total RNA was separated on a 1.5% agarose gel, transferred to a positively charged nitrocellulose membrane (Hybond-XL, Amersham Bioscience) and exposed to a labeled probe. The radioactive signal was visualized on Fuji X-ray film. The DNA probe for the *RDN18* gene was a complete ORF of the gene prepared by PCR reaction. For the *FLO11* probe, a PCR fragment corresponding to the last 1382 bp of the *FLO11* gene was used. The [α-^32^P] dCTP-labeled probes were obtained by random priming using the DecaPrime II Kit (Ambion).

### Microarray analysis

The mRNA was isolated from total RNA using a Micro-Fast track 2.0 Kit (Invitrogen). Reverse transcription was performed from 2–4 μg of mRNA as previously described [36]. Labeled cDNA was applied to Yeast 6.4 K Array (Y6.4 K) double spotted ORF slides (University Health network, Toronto, Canada) following the manufacturer’s instructions and incubated overnight at 37°C. The arrays were washed according to the manufacturer’s protocol and scanned using an Axon fluorescent scanner and the GenePix software. Three double-genome microarrays (biological replicates) were used. Spots were detected using the TIGR Spotfinder (TM4.org) software and normalized in TIGR MIDAS (TM4.org) using the LOWESS method. Differentially expressed genes were selected using a combined fold-change and ANOVA analysis using MeV software (TM4.org). Genes exhibiting an at least 1.5-fold change (log_2_ > 0.585) in their average expression and simultaneously a p-value < 0.05 were considered to be differentially expressed.

### Extracellular material extraction, SDS-PAGE and detection of protein glycosylation

Extracellular material was extracted as previously described [14]. After SDS-PAGE separation, extracellular proteins were stained with silver [37] or transferred to a PVDF membrane. Glycoproteins were visualized by sequential incubation in concanavalin A, horseradish peroxidase and chloronaphthol H_2_O_2_ solution [38].

### Two-photon excitation confocal microscopy (2P-CM)

Vertical transverse cross-sections of colonies were prepared and their side-views obtained by 2P-CM as described previously [39]. The cross-sections were stained with Concanavalin A conjugated with Alexa Fluor 488 (ConA-AF; 30 μg/ml). Alternatively, GFP fluorescence was monitored. Images were acquired with a Leica TCS SP2 AOBS MP confocal laser scanning microscope fitted with a mode-locked Ti: Sapphire Chameleon Ultra laser (Coherent Inc., Santa Clara, CA, USA) for two-photon excitation as previously described [16].

### Real time quantitative PCR (qPCR) analysis of genomic DNA

Three target genes were selected from those in the left arm of chromosome XV with apparently upregulated expression in strain BR-RF compared with BR-S and three reference genes were selected from those in the right arm of chromosome XV with no significant difference in expression (Figure 5). Primers (Additional file 2: Table S4) were designed using the GenScript real time qPCR primer design tool (https://www.genscript.com/ssl-bin/app/primer). Stipulated parameters included an amplicon length of 90–110 bp and melting temperature of 60-65°C. Primers were selected with a GC content of 40–60%, a length of 19–21 bp and minimal self-complementarity when analyzed using OligoCalc (http://www.basic.northwestern.edu/biotools/oligocalc.html[40]). DNA extraction was carried out according to the method of [41]. Real time qPCR was carried out on the light cycler 480 II (Roche, Basel) using FAST START Sybr Green (Roche, Basel), forward and reverse primers (final concentration 500 nM) and genomic DNA (20 ng per 20 μl reaction) from strain BR-RF or strain BR-S (or water in negative controls). An initial melting/activation step at 95°C for 5 min was followed by 45 cycles of melting, annealing and amplification (95°C for 30 s, 56°C for 30 s and 72°C for 30 s) with a fluorescence measurement (465–510 nm) at the end of each amplification step. Raw data were analyzed using the comparative delta CT method [42]. The relative gene copy number is 2^ΔCT^, where ΔCT = (CT_BR-S_-CT_BR-RF_).

### In silico analysis of gene sets obtained by microarray comparison

Genes were classified into functional categories using the particular gene description in the *Saccharomyces* genome database available at http://www.yeastgenome.org/. Enrichment of the gene sets at particular chromosomal regions was determined by Positional Gene Enrichment analysis using the online tool available at http://homes.esat.kuleuven.be/~bioiuser/pge[43].

## Availability of supporting data

The data sets supporting the results of this article are available in the NCBI GEO repository (accession number GSE40625, http://www.ncbi.nlm.nih.gov/geo/query/acc.cgi?acc=gse40625).

## Competing interests

The authors declare that they have no competing interests.

## Authors’ contributions

ZP, LV, VS and DW conceived and designed the study. VS, MB, DW, ZP and LV performed the experiments. VS, ZP, LV, DW and MB analyzed the data. ZP, LV, VS and DW wrote the paper. All authors read and approved the final manuscript.

## Supplementary Material

Additional file 1: Table S1 List of genes differentially expressed comparing transcriptomes of cells from 4- and 7- day-old BR-F, BR-RF and BR-S colonies. Differentially expressed genes were selected on the basis of combined criteria of fold-change (0.585 on log2 scale) and p-value (<0.05). Gene sets obtained from comparison of each phenotype couple (BR-F x BR-S, BR-F x BR-RF and BR-S x BR-RF) for both 4- and 7-day-old colonies are on a separate xls list. Click here for file

Additional file 2: Table S2 List of strains used in this study. **Table S3.** List of primers and plasmids used in the strain construction. **Table S4.** List of primers used in qPCR. Click here for file

## References

[B1] Palková Z Multicellular microorganisms: laboratory versus nature EMBO Rep 2004 5 5 470 476 10.1038/sj.embor.7400145 15184977 PMC1299056

[B2] van der Woude MW Re-examining the role and random nature of phase variation FEMS Microbiol Lett 2006 254 2 190 197 10.1111/j.1574-6968.2005.00038.x 16445745

[B3] Soll DR High-frequency switching in *Candida albicans* Clin Microbiol Rev 1992 5 2 183 203 1576587 10.1128/cmr.5.2.183 PMC358234

[B4] Soll DR *Candida* commensalism and virulence: the evolution of phenotypic plasticity Acta Trop 2002 81 2 101 110 10.1016/S0001-706X(01)00200-5 11801217

[B5] Jain N Hasan F Fries BC Phenotypic switching in fungi Curr Fungal Infect Rep 2008 2 3 180 188 10.1007/s12281-008-0026-y 19768140 PMC2746697

[B6] Fries BC Taborda CP Serfass E Casadevall A Phenotypic switching of *Cryptococcus neoformans* occurs in vivo and influences the outcome of infection J Clin Invest 2001 108 11 1639 1648 10.1172/JCI200113407 11733559 PMC200988

[B7] Kvaal C Lachke SA Srikantha T Daniels K McCoy J Soll DR Misexpression of the opaque-phase-specific gene *PEP1* (SAP1) in the white phase of *Candida albicans* confers increased virulence in a mouse model of cutaneous infection Infect Immun 1999 67 12 6652 6662 10569787 10.1128/iai.67.12.6652-6662.1999 PMC97079

[B8] Goldman DL Fries BC Franzot SP Montella L Casadevall A Phenotypic switching in the human pathogenic fungus *Cryptococcus neoformans* is associated with changes in virulence and pulmonary inflammatory response in rodents Proc Natl Acad Sci USA 1998 95 25 14967 14972 10.1073/pnas.95.25.14967 9843999 PMC24559

[B9] Slutsky B Staebell M Anderson J Risen L Pfaller M Soll DR “White-opaque transition”: a second high-frequency switching system in *Candida albicans* J Bacteriol 1987 169 1 189 197 3539914 10.1128/jb.169.1.189-197.1987 PMC211752

[B10] Ramirez-Zavala B Reuss O Park YN Ohlsen K Morschhauser J Environmental induction of white-opaque switching in *Candida albicans* PLoS Pathog 2008 4 6 e1000089 10.1371/journal.ppat.1000089 18551173 PMC2405950

[B11] Granek JA Magwene PM Environmental and genetic determinants of colony morphology in yeast PLoS Genet 2010 6 1 e1000823 10.1371/journal.pgen.1000823 20107600 PMC2809765

[B12] Granek JA Murray D Kayrkci O Magwene PM The genetic architecture of biofilm formation in a clinical isolate of *Saccharomyces cerevisiae* Genetics 2013 193 2 587 600 10.1534/genetics.112.142067 23172850 PMC3567746

[B13] Kuthan M Devaux F Janderová B Slaninová I Jacq C Palková Z Domestication of wild *Saccharomyces cerevisiae* is accompanied by changes in gene expression and colony morphology Mol Microbiol 2003 47 3 745 754 10.1046/j.1365-2958.2003.03332.x 12535073

[B14] Šťovíček V Váchová L Kuthan M Palková Z General factors important for the formation of structured biofilm-like yeast colonies Fungal Genet Biol 2010 47 12 1012 1022 10.1016/j.fgb.2010.08.005 20728557

[B15] Voordeckers K De Maeyer D van der Zande E Vinces MD Meert W Cloots L Ryan O Marchal K Verstrepen KJ Identification of a complex genetic network underlying *Saccharomyces cerevisiae* colony morphology Mol Microbiol 2012 86 1 225 239 10.1111/j.1365-2958.2012.08192.x 22882838 PMC3470922

[B16] Váchová L Šťovíček V Hlaváček O Chernyavskiy O Štěpánek L Kubínová L Palková Z Flo11p, drug efflux pumps, and the extracellular matrix cooperate to form biofilm yeast colonies J Cell Biol 2011 194 5 679 687 10.1083/jcb.201103129 21875945 PMC3171128

[B17] Vopálenská I Šťovíček V Janderová B Váchová L Palková Z Role of distinct dimorphic transitions in territory colonizing and formation of yeast colony architecture Environ Microbiol 2010 12 1 264 277 10.1111/j.1462-2920.2009.02067.x 19799621

[B18] Srikantha T Tsai L Daniels K Klar AJ Soll DR The histone deacetylase genes *HDA1* and *RPD3* play distinct roles in regulation of high-frequency phenotypic switching in *Candida albicans* J Bacteriol 2001 183 15 4614 4625 10.1128/JB.183.15.4614-4625.2001 11443097 PMC95357

[B19] Perez-Martin J Uria JA Johnson AD Phenotypic switching in *Candida albicans* is controlled by a *SIR2* gene EMBO J 1999 18 9 2580 2592 10.1093/emboj/18.9.2580 10228170 PMC1171338

[B20] Bedalov A Gatbonton T Irvine WP Gottschling DE Simon JA Identification of a small molecule inhibitor of Sir2p Proc Natl Acad Sci USA 2001 98 26 15113 15118 10.1073/pnas.261574398 11752457 PMC64992

[B21] Aparicio OM Billington BL Gottschling DE Modifiers of position effect are shared between telomeric and silent mating-type loci in S. cerevisiae Cell 1991 66 6 1279 1287 10.1016/0092-8674(91)90049-5 1913809

[B22] Robyr D Suka Y Xenarios I Kurdistani SK Wang A Suka N Grunstein M Microarray deacetylation maps determine genome-wide functions for yeast histone deacetylases Cell 2002 109 4 437 446 10.1016/S0092-8674(02)00746-8 12086601

[B23] Alby K Bennett RJ Stress-induced phenotypic switching in *Candida albicans* Mol Biol Cell 2009 20 14 3178 3191 10.1091/mbc.E09-01-0040 19458191 PMC2710840

[B24] Verstrepen KJ Reynolds TB Fink GR Origins of variation in the fungal cell surface Nat Rev Microbiol 2004 2 7 533 540 10.1038/nrmicro927 15197389

[B25] Fidalgo M Barrales RR Jimenez J Coding repeat instability in the *FLO11* gene of *Saccharomyces* yeasts Yeast 2008 25 12 879 889 10.1002/yea.1642 19160455

[B26] Rinckel LA Garfinkel DJ Influences of histone stoichiometry on the target site preference of retrotransposons Ty1 and Ty2 in *Saccharomyces cerevisiae* Genetics 1996 142 3 761 776 8849886 10.1093/genetics/142.3.761 PMC1207017

[B27] Roncero C Duran A Effect of Calcofluor white and Congo red on fungal cell wall morphogenesis: in vivo activation of chitin polymerization J Bacteriol 1985 163 3 1180 1185 3897187 10.1128/jb.163.3.1180-1185.1985 PMC219256

[B28] Ram AF Wolters A Ten Hoopen R Klis FM A new approach for isolating cell wall mutants in *Saccharomyces cerevisiae* by screening for hypersensitivity to calcofluor white Yeast 1994 10 8 1019 1030 10.1002/yea.320100804 7992502

[B29] Klar AJ Srikantha T Soll DR A histone deacetylation inhibitor and mutant promote colony-type switching of the human pathogen *Candida albicans* Genetics 2001 158 2 919 924 11404352 10.1093/genetics/158.2.919 PMC1461676

[B30] Pavelka N Rancati G Zhu J Bradford WD Saraf A Florens L Sanderson BW Hattem GL Li R Aneuploidy confers quantitative proteome changes and phenotypic variation in budding yeast Nature 2010 468 7321 321 325 10.1038/nature09529 20962780 PMC2978756

[B31] Pfau SJ Amon A Chromosomal instability and aneuploidy in cancer: from yeast to man EMBO Rep 2012 13 6 515 527 10.1038/embor.2012.65 22614003 PMC3367249

[B32] Tan Z Hays M Cromie GA Jeffery EW Scott AC Ahyong V Sirr A Skupin A Dudley AM Aneuploidy underlies a multicellular phenotypic switch Proc Natl Acad Sci USA 2013 110 30 12367 12372 10.1073/pnas.1301047110 23812752 PMC3725063

[B33] Slutsky B Buffo J Soll DR High-frequency switching of colony morphology in *Candida albicans* Science 1985 230 4726 666 669 10.1126/science.3901258 3901258

[B34] Slavikova E Vadkertiova R Yeasts and yeast-like organisms isolated from fish-pond waters Acta Microbiol Pol 1995 44 2 181 189 8906934

[B35] Gietz RD Woods RA Transformation of yeast by lithium acetate/single-stranded carrier DNA/polyethylene glycol method Methods Enzymol 2002 350 87 96 12073338 10.1016/s0076-6879(02)50957-5

[B36] Cap M Stepanek L Harant K Vachova L Palkova Z Cell differentiation within a yeast colony: metabolic and regulatory parallels with a tumor-affected organism Mol Cell 2012 46 4 436 448 10.1016/j.molcel.2012.04.001 22560924

[B37] Rabilloud T Vuillard L Gilly C Lawrence JJ Silver-staining of proteins in polyacrylamide gels: a general overview Cell Mol Biol (Noisy-le-grand) 1994 40 1 57 75 8003936

[B38] Hawkes R Identification of concanavalin a-binding proteins after sodium dodecyl sulfate–gel electrophoresis and protein blotting Anal Biochem 1982 123 1 143 146 10.1016/0003-2697(82)90634-0 7114467

[B39] Váchová L Chernyavskiy O Strachotová D Bianchini P Burdíková Z Ferčíková I Kubínová L Palková Z Architecture of developing multicellular yeast colony: spatio-temporal expression of Ato1p ammonium exporter Environ Microbiol 2009 11 1866 1877 10.1111/j.1462-2920.2009.01911.x 19302539

[B40] Kibbe WA OligoCalc: an online oligonucleotide properties calculator Nucleic Acids Res 2007 35 Web Server issue W43 W46 17452344 10.1093/nar/gkm234 PMC1933198

[B41] Harju S Fedosyuk H Peterson KR Rapid isolation of yeast genomic DNA: Bust n’ Grab BMC Biotechnol 2004 4 8 10.1186/1472-6750-4-8 15102338 PMC406510

[B42] Pfaffl MW A new mathematical model for relative quantification in real-time RT-PCR Nucleic Acids Res 2001 29 9 e45 10.1093/nar/29.9.e45 11328886 PMC55695

[B43] De Preter K Barriot R Speleman F Vandesompele J Moreau Y Positional gene enrichment analysis of gene sets for high-resolution identification of overrepresented chromosomal regions Nucleic Acids Res 2008 36 7 e43 10.1093/nar/gkn114 18346969 PMC2367735

