# Supplementary material for: Global changes in gene expression associated with phenotypic switching of wild yeast
Source: BMC Genomics. 2014 Feb 17;15:136. doi: 10.1186/1471-2164-15-136 (PMC3930820; doi:10.1186/1471-2164-15-136)
Supplement: Additional file 2: Table S2 — List of strains used in this study. Table S3. List of primers and plasmids used in the strain construction. Table S4. List of primers used in qPCR. [file 1471-2164-15-136-S2.pdf]

Table S2. **List of strains used in this study**.

| strain | genotype | reference |
|---|---|---|
| BR-F | MATa/MATα | (Kuthan et al., 2003) |
| BR-S | MATa/MATα | (Kuthan et al., 2003) |
| BR-F-RF | MATa/MATα | this study |
| BR-F-Flo11p-GFP | MATa/MATα *FLO11*/*FLO11*-EGFP | this study |
| BR-F-RF-Flo11p-GFP | MATa/MATα *FLO11*/*FLO11*-EGFP | this study |
| BR-F-RF-$p_{GAL1}$-GFP | MATa/MATα *HIS3*/*his3Δ*::nat1-$p_{GAL1}$-GFP | this study |
| BR-F-*sir2* | MATa/MATα *sir2Δ*::kanMX/*sir2Δ*::nat1 | this study |
| BR-S-*hda1* | MATa/MATα *hda1Δ*::kanMX/*hda1Δ*::nat1 | this study |

Table S3. **List of primers and plasmids used in the strain construction.**

| primer | sequence | purpose |
|---|---|---|
| **SIR2-del-for** | AGACACATTCAAACCATTTTTCCCTCATCGGCACATT AAAGCTGGCAGCTGAAGCTTCGTACGC | deletion of *SIR2* gene |
| **SIR2-del-rev** | ATTGATATTAATTTGGCACTTTTAAATTATTAAATTGC CTTCTACGCATAGGCCACTAGTGGATCTG | deletion of *SIR2* gene |
| **HDA1-del-for** | GAGAAAGGGAAAGTTGAGCACTGTAATACGCCGAAC AGATTAAGCCAGCTGAAGCTTCGTACGC | deletion of *HDA1* gene |
| **HDA1-del-rev** | TTCTTCATCACTCCATTCTTCAAACGAATCCAGTATA AAGTCTGTGCATAGGCCACTAGTGGATCTG | deletion of *HDA1* gene |
| **FLO11-eGFP-for** | GCTTATTTGGTCCTTTCGCTTCTATTTAACTCGGCTT TGGGTTTTTGCAGGTCGACAACCCTTAAT | internal fusion of GFP with *FLO11* gene |
| **FLO11-eGFP-rev** | ACAGCTAGTTCCTTCGGAGGATCCTCTTGGAACTAG TGCAGTTGGGCGGCCGCATAGGCCACT | internal fusion of GFP with *FLO11* gene |
| **$P_{GAL1}$-eGFP-for** | CTTCGAAGAATATACTAAAAAATGAGCAGGCAAGATA AACGAAGGCAAAGATGCGTACGCTGCAGGTCGAC | $P_{GAL1}$-GFP cassette for genomic integration |
| **$P_{GAL1}$-eGFP-rev** | TATACACATGTATATATATCGTATGCTGCAGCTTTAA ATAATCGGTGTCACTACGATGAATTCTCTGTCG | $P_{GAL1}$-GFP cassette for genomic integration |

| plasmid | application | reference |
|---|---|---|
| pAG25 | deletion cassette (nat marker) | (Goldstein and McCusker, 1999) |
| pUG6 | deletion cassette (kanMX marker) | (Gueldener et al., 2002) |
| pYM-N25 | $P_{GAL1}$-GFP cassette | (Janke et al., 2004) |
| pOM40 | internal GFP fusion | (Gauss et al., 2005) |
| pSH65 | marker excision | (Gauss et al., 2005) |

Table S4. **List of primers used in qPCR**.

| Primer | ORF | Gene | Sequence | Tm | % GC | nt |
|---|---|---|---|---|---|---|
| MAM3Fd | YOL060C | *MAM3* | tctagggatgttgccgattc | 64.3 | 50 | 20 |
| MAM3Rv | YOL060C | *MAM3* | tacaccacccagcagcacta | 64.8 | 55 | 20 |
| SHR5Fd | YOL110W | *SHR5* | cgtctcgcctctgtcagaat | 65 | 55 | 20 |
| SHR5Rv | YOL110W | *SHR5* | ccgaacacctcaccttcttc | 64.1 | 55 | 20 |
| TOA1Fd | YOR194C | *TOA1* | acaaaggcgagatggaaatg | 64.3 | 45 | 20 |
| TOA1Rv | YOR194C | *TOA1* | tatacccactccgcttccac | 64.2 | 55 | 20 |
| CLP1Fd | YOR250C | *CLP1* | gtcaaggtaaactcgggcat | 63.2 | 50 | 20 |
| CLP1Rv | YOR250C | *CLP1* | tcgacagcgtatatgggaaa | 63.4 | 45 | 20 |
| MPD1Fd | YOR288C | *MPD1* | agcttcgataaagcgatcca | 64 | 45 | 20 |
| MPD1Rv | YOR288C | *MPD1* | gccttgcggaacgtactaga | 64.5 | 55 | 20 |

Fd: forward primer, Rv: reverse primer, Tm: melting temperature, nt: nucleotides

**Supplemental references**

Gauss, R., Trautwein, M., Sommer, T., and Spang, A. (2005) New modules for the repeated internal and N-terminal epitope tagging of genes in Saccharomyces cerevisiae. *Yeast* **22**: 1-12.

Goldstein, A.L., and McCusker, J.H. (1999) Three new dominant drug resistance cassettes for gene disruption in Saccharomyces cerevisiae. *Yeast* **15**: 1541-1553.

Gueldener, U., Heinisch, J., Koehler, G.J., Voss, D., and Hegemann, J.H. (2002) A second set of loxP marker cassettes for Cre-mediated multiple gene knockouts in budding yeast. *Nucleic Acids Res* **30**: e23.

Janke, C., Magiera, M.M., Rathfelder, N., Taxis, C., Reber, S., Maekawa, H. et al. (2004) A versatile toolbox for PCR-based tagging of yeast genes: new fluorescent proteins, more markers and promoter substitution cassettes. *Yeast* **21**: 947-962.

Kuthan, M., Devaux, F., Janderová, B., Slaninová, I., Jacq, C., and Palková, Z. (2003) Domestication of wild Saccharomyces cerevisiae is accompanied by changes in gene expression and colony morphology. *Mol Microbiol* **47**: 745-754.
